# Remyelination-Promoting DNA Aptamer Conjugate Myaptavin-3064 Binds to Adult Oligodendrocytes In Vitro

**DOI:** 10.3390/ph13110403

**Published:** 2020-11-19

**Authors:** Mahboubeh Fereidan-Esfahani, Wei Ying Yue, Brandon Wilbanks, Aaron J. Johnson, Arthur E. Warrington, Charles L. Howe, Moses Rodriguez, Louis J. Maher

**Affiliations:** 1 Department of Neurology, Mayo Clinic, Rochester, MN 55905, USA; Fereidan-Esfahani.Mahboobeh@mayo.edu (M.F.-E.); yue.weiying@mayo.edu (W.Y.Y.); warrington.arthur@mayo.edu (A.E.W.); howe@mayo.edu (C.L.H.); rodriguez.moses@mayo.edu (M.R.); 2 Department of Biochemistry and Molecular Biology, Mayo Clinic, Rochester, MN 55905, USA; wilbanks.brandon@mayo.edu; 3 Department of Immunology, Mayo Clinic, Rochester, MN 55905, USA; Johnson.Aaron2@mayo.edu; 4 Department of Neurologic Surgery, Mayo Clinic, Rochester, MN 55905, USA

**Keywords:** multiple sclerosis, DNA aptamer, human oligodendroglial cell line, oligodendrocytes, oligodendrocyte progenitor cells (OPCs)

## Abstract

We previously applied Systematic Evolution of Ligands by EXponential enrichment (SELEX) technology to identify myelin-specific DNA aptamers, using crude mouse central nervous system myelin as bait. This selection identified a 40-nucleotide aptamer (LJM-3064). Multiple biotinylated LJM-3064 molecules were conjugated to a streptavidin core to mimic a multimeric immunoglobulin M (IgM) antibody, generating 3064-BS-streptavidin (Myaptavin-3064). We previously showed that Myaptavin-3064 induces remyelination in the Theiler’s murine encephalomyelitis virus (TMEV) model of chronic spinal cord demyelination. While details of target binding and the mechanism of action remain unclear, we hypothesized that Myaptavin-3064 induces remyelination by binding to oligodendrocytes (OLs). We now report the results of binding assays using the human oligodendroglioma (HOG) cell line, applying both flow cytometry and immunocytochemistry (IC) to assay aptamer conjugate binding to cells. IC assays were applied to compare aptamer conjugate binding to primary embryonic mouse mixed cortical cultures and primary adult rat mixed glial cultures. We show that Myaptavin-3064 binds to HOG cells, with increased binding upon differentiation. In contrast, a negative control aptamer conjugate, 3060-BS, which did not promote central nervous system (CNS) remyelination, does not bind to HOG cells. Myaptavin-3064 did not bind to lung (L2) or kidney (BHK) cell lines. Total internal reflection fluorescence (TIRF) imaging indicates that Myaptavin-3064 binds at the cell membrane of live cells. In addition to HOG cells, Myaptavin-3064 binds to adult rat OLs, but not to embryonic mouse mixed cortical cultures. These data support the hypothesis that Myaptavin-3064 binds to a surface molecule on both rodent and human OLs in a manner that triggers a remyelination signal pathway.

## 1. Introduction

Multiple sclerosis (MS) is a chronic demyelinating disease of the central nervous system (CNS), typically beginning as a relapsing-remitting disease (RRMS) [1]. MS is thought to initiate with inflammation of unknown etiology, which leads to focal demyelination in the CNS, and is treated with a wide range of non-curative immunosuppressive agents [2]. Even in actively-treated patients, 36% of cases evolve into a progressive phase [3]. In pathologically inactive progressive cases, inflammation and axonal injury diminish to levels seen in age-matched controls, but neurodegeneration continues [4]. The remyelinating capacity of the CNS is apparently exhausted, leading to further progression and disability. Anti-inflammatory drugs are usually not beneficial in this stage.

Inspired by the identification of natural immunoglobulin M (IgM) antibodies capable of stimulating therapeutic remyelination [5], we launched an effort to identify alternative remyelinating agents mimicking antibody functions, but with smaller size, enhanced ability to cross the blood–brain barrier (BBB), improved half-life, and lower cost. As the identified remyelinating IgMs generally show reactivity to myelin and oligodendrocytes (OLs), we previously used Systematic Evolution of Ligands by EXponential enrichment (SELEX) [6] technology to identify myelin-specific DNA aptamers using murine crude myelin as the selection target [7]. This selection identified aptamer LJM-3064 as a 40-nucleotide guanosine-rich anti-myelin DNA aptamer capable of adopting G-quadruplex structures. In its monomeric form, LJM-3064 does not promote remyelination. Terminally biotinylated LJM-3064 can be conjugated with streptavidin to create multimers reminiscent of multivalent antibodies. This streptavidin conjugate is termed Myaptavin-3064. Previous studies of DNA aptamers that bind to immunoglobulin M (IgM), thrombin and VEGF have confirmed the fact that linking multiple aptamer copies can lead to a multivalency effect that improves affinity and the potential for target cross-linking without compromising specificity [8,9,10,11].

Intraperitoneal injection of Myaptavin-3064 into mice with chronic spinal cord demyelination by infection with Theiler’s murine encephalomyelitis virus (TMEV) results in aptamer conjugate distribution into CNS tissue, enhancing remyelination in 35% of experimental CNS sections compared to 9% for negative control molecule, 3060-BS [7]. Agents that trigger regeneration of oligodendrocytes by direct binding to these cells presumably must gain access by crossing the blood–brain barrier. This penetration may be facilitated in MS by inflammation and lesions that compromise the normal structure of this barrier. We previously studied this issue in normal mice and animals with TMEV infection [12]. Pharmacokinetics studies using quantitative polymerase chain reaction (qPCR) demonstrated that Myaptavin-3064 has broad and rapid tissue distribution including accumulation in the brain and spinal cord of both healthy and TMEV-infected animals. The mechanism of action of Myaptavin-3064 remains unclear. A logical step towards understanding the precise biological mechanism is to determine relevant target cells. Therefore, we searched for CNS targets of Myaptavin-3064.

## 2. Results

### 2.1. Characterization of HOG Cells in Culture

**Figure 1:**
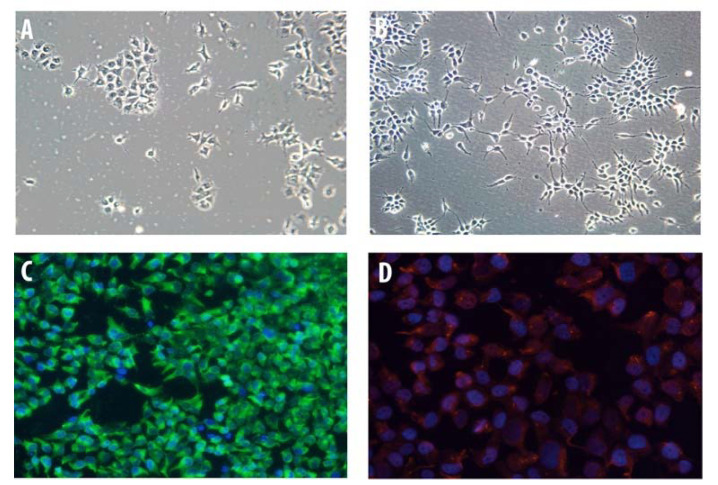
Human oligodendroglioma (HOG) cell characterization. HOG cells were grown on poly-L-lysine-coated cover slips for five days. (**A**) Undifferentiated HOG cells showed a flat and epithelioid-like morphology with large round nuclei and short processes. (**B**) After two days in differentiation media, HOG cells became more mature with a fried-egg phenotype, spindle-shaped nuclei, and long processes. (**C**) For immunocytochemical characterizations, differentiated HOG cells were fixed, permeabilized, and stained for myelin basic protein (MBP) followed by fluorescein-5-isothiocyanate (FITC) secondary antibody. (**D**) Cells were stained for 2′,3′-cyclic nucleotide-3′-phosphodiesterase (CNPase). Immunocytochemical characterization of differentiated HOG cells showed that most cells expressed (**C**) MBP and (**D**) CNPase.

**Figure 2:**
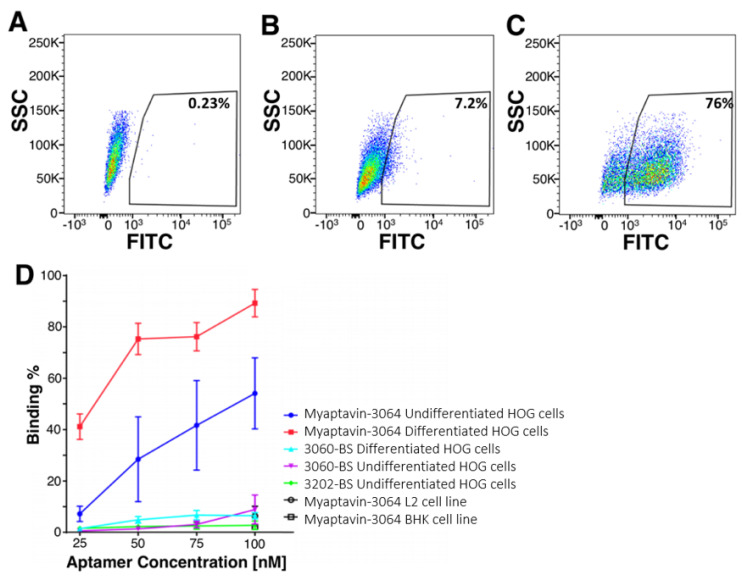
Adherence of Myaptavin-3064 to HOG cells measured by flow cytometry. Shown are flow cytometric signals from HOG cells incubated with no aptamer (**A**), 50 nM 3060-BS negative control aptamer conjugate (**B**), or 50 nM Myaptavin-3064 conjugate (**C**). Gating was set with reference to staining with negative control 3060 BS 50 nM aptamer conjugate. Concentration-dependent binding studies (**D**) show binding of Myaptavin-3064 to undifferentiated HOG cells (blue line with filled circles), differentiated HOG cells (red line, filled squares) compared to 3060-BS binding to either differentiated (cyan line, filled triangle) HOG cells or undifferentiated HOG (magenta closed triangle). Myaptavin-3064 binding to L2 cells (black line open circle) and to BHK cells (black line open squares) are also shown. SCC: side scatter channel; FITC: fluorescein-5-isothiocyanate.

Undifferentiated human oligodendroglioma (HOG) cells showed a flat and epithelioid-like morphology with large round nuclei and short processes that had limited resemblance to primary cultured OLs (Figure 1A). In contrast, upon culture in differentiation medium, cells exhibited a more mature OL-like morphology with spindle-shaped nuclei and long processes (Figure 1B). Immunocytochemistry (IC) of fixed HOG cells showed that nearly all cells expressed myelin basic protein (MBP) (Figure 1C) and 2′,3′-cyclic nucleotide-3′-phosphodiesterase (CNPase) (Figure 1D).

### 2.2. Specific Binding of Myaptavin-3064 to HOG Cells

Figure 2 depicts fluorescent aptamer conjugate binding to HOG cells as determined by flow cytometry. Data for a population of HOG cells incubated with no aptamer are shown in Figure 2A, compared to 50 nM 3060-BS (Figure 2B), and Myaptavin-3064 (Figure 2C). Gating with 3060-BS was used as a reference. Concentration-dependent flow cytometry measurements using anti-streptavidin staining demonstrated that nearly 50% of undifferentiated HOG cells could be recognized by Myaptivin-3064 (Figure 2D). The fraction of HOG cells bound by Myaptivin-3064 increased in a dose-dependent manner from less than 10% at 25 nM to more than 50% at 100 nM. Using differentiated HOG cells, nearly 90% bound Myaptivin-3064 at the highest tested concentration of 100 nM. The negative control, 3060-BS, bound few HOG cells of either differentiation state at any tested concentration. Additional flow cytometry analysis was performed using a variety of cell lines such as BHK-21 and L-2 to assay aptamer conjugate specificity. Myaptavin-3064 bound fewer than 10% of BHK-21 and L-2 cell lines, i.e., no better than the negative controls (Figure 2D).

**Figure 3:**
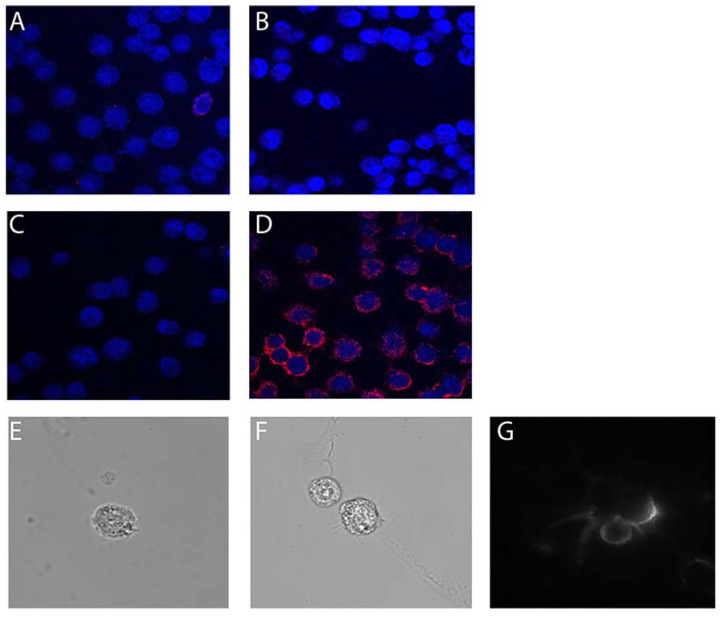
Immunofluorescence staining of undifferentiated HOG cells by 3064-BS (Myaptavin-3064). Live staining was performed on unfixed cells using (**A**) 100 nM 3060-BS, (**B**) 100 nM 3202-BS, (**C**) no aptamer conjugate, and (**D**) 100 nM Myaptavin-3064 for 1 h on ice. After washing, cells were fixed and stained with labeled anti-streptavidin antibodies and DAPI counterstaining of nuclei. Similar staining methods were applied to 1.5 mm poly-L-lysine-coated cover slips to determine cell surface binding of Myaptavin-3064 to HOG cells. There was no signal from negative control 3060-BS (**E**) or in the absence of aptamer conjugate (**F**). Myaptavin-3064 binds to the cell membrane of the HOG cells after 15 min of incubation as detected by total internal reflection fluorescence (TIRF) microscopy using live cell staining (**G**).

IC staining of undifferentiated live HOG cells confirmed our flow cytometry data (Figure 3). While undifferentiated HOG cells were negative for binding to negative control conjugates 3060-BS (Figure 3A) and 3202-BS (Figure 3B), compared to no conjugate (Figure 3C), Myaptavin-3064 bound to almost 100% of differentiated HOG cells (Figure 3D). Similar staining methods were applied to examine the binding of aptamers to undifferentiated HOG cells by total internal reflection (TIRF) microscopy. Negative control 3060-BS did not bind detectably (Figure 3E) compared to unstained (Figure 3F), whereas Myaptavin-3064 bound to the membrane of HOG cells (Figure 3G).

### 2.3. Myaptavin-3064 Cellular Targets

**Figure 4:**
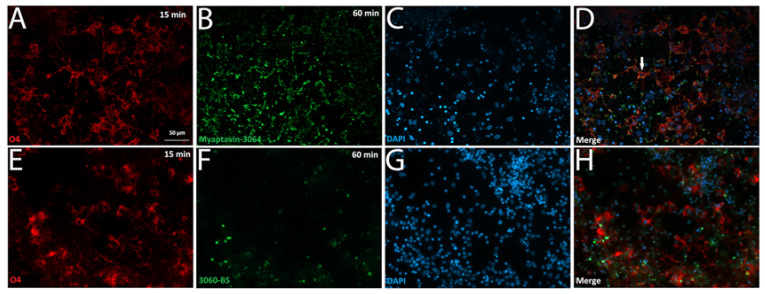
Aptamer conjugate binding to adult rat oligodendrocytes. Mixed glial cells obtained from adult rat brain tissue were grown on laminin- and poly-ornithine-coated cover slips for three weeks. Live staining was performed on unfixed cells after incubation for 15 min with O4 (**A**,**E**), DAPI (**B**,**F**) and for 60 min with Myaptavin-3064 (**C**,**D**) or 3060-BS (**G**,**H**). Most O4-positive OLs were positive for Myaptavin-3064 binding (**C**,**D**), whereas minimal binding was observed for negative control aptamer conjugate 3060-BS (**G**,**H**). The white arrow in (**D**) indicates an example of co-localization of O4 (red) and Myaptavin-3064 (green) on adult rat oligodendrocytes (OLs).

We tested aptamer conjugate binding to primary cortical cultures obtained from an embryonic mouse and found no aptamer co-staining with antibodies to O4, beta-III tubulin, or glial fibrillary acidic protein (GFAP) (Supplementary Figure S1). However, when a similar staining method was applied to test aptamer binding to primary cultures obtained from adult rat tissue (Figure 4), 97% of cells positive for both O4 staining (Figure 4A,E) and DAPI staining (Figure 4B,F) were positive for binding to Myaptavin-3064 (Figure 4C,D) but not to control conjugate LJM-3060BS (Figure 4G,H). Confocal imaging shows that Myaptavin-3064 binds to the cell membrane of most O4-positive OLs (Figure 4C,D) compared to nuclear staining of a few dead cells by 3060-BS, which may occur by nonspecific mechanisms.(Figure 4G,H).

## 3. Discussion

We present new evidence that remyelinating DNA aptamer conjugate Myaptavin-3064 binds to the surface of HOG cells and OLs obtained from an adult rat brain. TIRF images indicate that the aptamer is localized to the HOG cell membrane within 15 min of treatment. This finding is consistent with previous reports that have shown that oligonucleotides do not freely penetrate the cell membrane [13]. Our flow cytometry results suggest that the antigen for Myapatavin-3064 is expressed on a greater fraction of differentiated HOG cells than undifferentiated cells, and we demonstrate binding to be dose-dependent.

Treatment of cortical cultures from embryonic mice with Myaptavin-3064 indicated low binding. As myelination occurs primarily after birth, this result suggests that the target of Myaptavin-3064 is expressed in more mature myelin [7].

Interestingly, a recent study by Shamili and colleagues [14] confirms and extends the observed efficacy of remyelinating aptamer LJM-3064. This group used a formulation of LJM-3064 based on mesenchymal stem cell-derived exosomes as a drug delivery system. They showed that LJM-3064-exosome bioconjugate could promote the proliferation of the oligodendroglioma cell line (OLN93) in vitro. Moreover, in vivo administration of the LJM-3064-exosome conjugate led to robust remyelination, suppression of inflammation, and improved disease severity in the mouse experimental autoimmune encephalomyelitis (EAE) model. This independent study points to the importance of future work to clarify the mechanism of formulations of LJM-3064. The observations reported here—that differentiated HOG cells are more strongly bound by LJM-3064 conjugates than undifferentiated HOG cells—provide a valuable new tool in this process.

CNS remyelination is believed to be a critical regenerative process that restores function and protects from axonal degeneration in MS and other demyelinating diseases [15,16,17]. It has been reported since 1970 that myelin repair requires resident oligodendrocyte progenitor cells (OPC)s to divide and repopulate a lesion before differentiating to mature OLs [18,19]. The role of OPCs in MS was further highlighted by showing the migration of glial progenitors in the subventricular zone of lesions in human postmortem brains [20]. Although mature adult OLs appear not to be involved in rodent CNS remyelination after toxin-induced spinal cord demyelination [21], a recent study has suggested the contribution of pre-existing adult OLs in remyelination in cats [16]. Two recent studies [22,23] have further highlighted the crucial role of mature OLs in remyelination in humans. Jakel and colleagues [23] used single-cell transcriptomics to analyze cells from normal appearing white matter (NAWM; defined as a region thought to be unaffected by focal inflammatory demyelination), chronic active, chronic inactive and remyelinated lesions within the same MS tissue block from patients. These authors suggest that morphologically normal OLs, even in NAWM, are actually affected by MS and are in an altered state. They also postulated that the number of OPCs was reduced both within MS plaques and in the NAWM. Interestingly, myelin gene expression was increased in mature OLs within lesions, leading the authors to question the general assumption that differentiation of resident OPCs to OLs is responsible for remyelination. In the study by Yeung et al. [22]), the authors analyzed the age of OLs in NAWM and shadow plaques consisting of thinly-myelinated axons (considered as evidence of incomplete remyelination in humans). They found that OLs in shadow plaques and lesions are actually as old as the patients. This suggests that these cells were not differentiated from newly recruited OPCs.

These results argue against the prevalent view that remyelination is mediated by adjacent OPCs migrating to demyelinated lesions, or adult neural stem cells residing in the subependymal zone [24,25]. Furthermore, the results emphasize the need to re-evaluate current regenerative strategies in MS, including OPC and stem cell transplantation. Myaptavin-3064 could be a unique candidate for remyelination therapy, as it exhibits exclusive reactivity to a human oligodendroglioma cell line and to OLs obtained from an adult brain.

**Figure 5:**
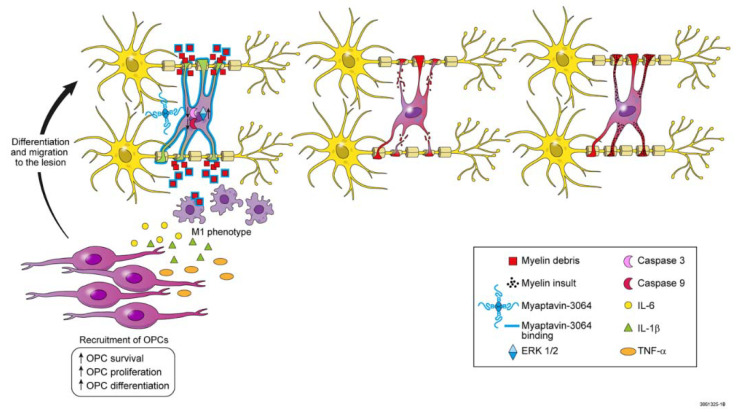
Illustration summarizing hypotheses for possible mechanisms of remyelination induced by Myaptavin-3064. *Direct mechanism:* Myaptavin-3064 binds to as-yet unknown membrane receptor(s) on post-mitotic OLs. Subsequently, conformational changes in the cell membranes of OLs trigger altered gene expression. For example, Myaptavin-3064 might mediate increased survival of adult OLs by reducing activation of caspase-3 and caspase-9 and increasing plasticity by activation of the extracellular signal-regulated kinase (ERK)1/2 pathway. *Indirect mechanism:* Given the ability of Myaptavin-3064 to bind central nervous system (CNS) myelin and as a result of the multimeric structure of this molecule, the agent might act as a “classical” immunoglobulin M (IgM), thereby opsonizing myelin debris and providing “find-me” and “eat-me” signals for microglia. M1 microglia are responsible for debris clearing in lesions. They also produce pro-inflammatory cytokines that might lead to recruitment, differentiation, and migration of OLs progenitor cells (OPC) into lesions.

At least two possible mechanisms could explain remyelination by Myaptavin-3064 (Figure 5). First, it is possible that Myaptavin-3064 binds to an as-yet unidentified membrane receptor on surviving adult OLs inducing conformational changes in OL cell membranes and activating the extracellular signal-regulated kinase (ERK)1/2 pathway to enhance remyelination by inducing plasticity and the extension of processes into lesions [26].

A second mechanism centers on the clearance of debris. It was previously shown that MS lesions contain substantial damaged myelin debris, which exerts an inhibitory effect on OPC differentiation [27,28]. Consistent with this “indirect” hypothesis, a recent study has shown enhanced phagocytosis of myelin debris in the presence of rHIgM22 [29]. It is thus possible that, given the ability of Myaptavin-3064 to bind CNS myelin, the conjugate acts as a “classical” IgM, opsonizing myelin debris and providing “find-me” and “eat-me” signals for microglia. Though M1 microglia are responsible for debris clearing in lesions, they also produce proinflammatory cytokines, which may intensify attacks [30]. Several studies have demonstrated that regulation of oligodendroglial development takes place, in large part, via factors that would otherwise be considered typical proinflammatory cytokines. IL-6 stimulates OPC survival and differentiation in vitro and knockout of cytokines impairs OPC differentiation and remyelination in vivo [31,32,33]. Additionally, the lack of TNFα led to a significant delay in remyelination and proliferation of OPCs [34]. Figure 5 illustrates future testable hypotheses for mechanisms by which Myaptavin-3064 might lead to remyelination following binding to OLs or myelin debris.

Current FDA-approved MS drugs focus on immunosuppression and do not prevent disease progression. These drugs do not promote remyelination. We believe that because MS lesions are diverse on a patient-to-patient basis, non-invasive markers for predicting lesion pathology would be profitable in MS research and care, opening the door to achievement of improved therapeutic outcomes by selecting suitable patients for remyelination therapies. We suggest that Myaptavin-3064 is a promising lead agent to block MS progression in patients with pattern I and II MS lesion pathologies where OLs remain.

## 4. Materials and Methods

### 4.1. Chemicals

Dulbecco’s modified Eagle Medium (DMEM) (10-017-CV), DMEM/F12 50:50 (10-090-CV), N2-supplement (17502-048), and penicillin/streptomycin (15140) were from Invitrogen (Carlsbad, CA, USA); Hank’s balanced salt solution (HBSS) (21-022-CV), 0.25% trypsin (25-050-CV) and sodium pyruvate (25-000-Cl) were from Mediatech (Manassas, VA, USA); fetal bovine serum (FBS) (SH30070.03) was from Hyclone (Waltham, MA, USA); FGF-2 (01–106) and PDGF-AA (01–309) was from Millipore (Temecula, CA, USA). Laminin was from R&D (Minneapolis, MN, USA). All other chemicals not specifically mentioned were purchased from Sigma (St. Louis, MO, USA).

### 4.2. Antibodies

Mouse monoclonal antibody IgM O4 was purified from hybridoma supernatant [35]. Commercial antibodies were CNPase (Sigma, C5922), MBP (Millipore, AB980), GFAP (Millipore, AB5804) and β-III tubulin (R&D MAB1195).

### 4.3. Preparation of Aptamer Complex

DNA aptamers were obtained from IDT (Coralville, IA, USA) with 3′ biotin-TEG modification as described previously (7). Then, 3′ biotinylated oligonucleotides were mixed in a 4:1 molar ratio with streptavidin to produce streptavidin-oligonucleotide conjugates. Remyelinating DNA aptamer is termed Myaptavin-3064 (5′ G_3_TCG_2_CG_3_TG_4_TG_3_AG_2_TG_2_TCT_2_GTCTCTG_3_T). Other conjugated and avidin complexed control oligonucleotides are identified using codes 3060-BS (5′ A_3_GA_2_CA_5_G_2_ATA_3_G_5_AGACG_6_A_2_CATG_4_) and 3202-BS (5′ T_40_).

### 4.4. Animals

Sprague Dawley rats and C57BL/6 mice were purchased from Harlan Laboratories (Madison, WI, USA) and Charles River Laboratories (Wilmington, MA, USA), respectively. All animal experiments were approved by the Mayo Clinic Institutional Animal Care and Use Committee through protocols A3057-17 approved on 11 February 2017 and A4102-18 approved on 11 September 2018.

### 4.5. Cell Culture

Cells were grown at 37 °C in a 5% CO_2_ environment in ambient oxygen. The human oligodendrocyte cell line (HOG) was provided by Dr. G. Dawson (University of Chicago, Chicago, IL, USA). Cells were cultured in growth medium containing DMEM supplemented with 10% FBS and 100 U/mL penicillin/streptomycin. To induce differentiation, two days after plating in flasks or on glass coverslips, 0.05% FBS, 30 nM triiodothyronine (T3), 30 nM selenium, 0.5 μg/mL insulin, 50 μg/mL transferrin and the same antibiotics were added to DMEM. Cell lines L2 (obtained from Dr. L. Pease, Mayo Clinic, Rochester, MN, USA) and BHK-21 (obtained from ATCC) were grown in DMEM supplemented with 5% and 10% FBS, respectively.

### 4.6. Isolation of Mouse Mixed Cortical Cells

Cerebral cortices were removed from C57BL6 mouse embryonic day 15 (E15) pups [36]. Meninges were removed and washed in HBSS. The tissue was washed in plating media containing high-glucose DMEM with glutamine, supplemented with 10% F12 and 10% FBS. Cortices were digested in 2 mg/mL papain in HBSS at 37 °C for 15 min. Cells were subjected to centrifugation at 400× *g* for 4 min and seeded at 4.5 × 10^5^ cells per cm^2^ on poly-ornithine coated plates. Three hours after seeding, cortical neurons were fed with neuron feeding media containing neurobasal media supplemented with 2% B27, 1% Glutamax, and 100 U/mL penicillin/streptomycin. Cells were exposed to 1 ng/mL BDNF and 10 ng/mL IGF-1 during the first 48 h. Cells were maintained in feeding media by changing half media volume every two days.

### 4.7. Adult Rat Mixed Glial Culture

Female Sprague Dawley rats (30 days of age) were subjected to aseptic cerebral cortex dissection and meninges were removed. Hemispheres were weighed, minced and transferred to Ca- and Mg-free HEPES-buffered Earle’s balanced salt solution (E/H) containing 5 g/L D-glucose, 3 g/L bovine serum albumin (BSA) fraction V, 20 mM HEPES and penicillin/streptomycin. The tissue was incubated in 0.1% trypsin, 10 µg/mL DNase I and 0.076% MgSO_4_ for 60 min at 37 °C in a rotary-shaking incubator at 130 rpm. Trypsin was inactivated with 10% FBS. The tissue was further dissociated by MgSO_4_ (0.0164% final concentration) and DNase I (20 mg/mL final concentration) and then incubated for 10 min on ice. The tissue was subjected to centrifugation in the cold at 850 rpm for 5 min. The pellet was re-suspended in E/H containing 10% FCS, 80 µg/mL DNase 1 and 0.076% MgSO_4_. Dissociated cells were filtered through 210 µm metal mesh, and the collected filtrate was passed twice through 60 µm nylon mesh. Sucrose gradient steps were used to isolate cells. The cell suspension was washed several times by centrifugation and plated on coverslips coated with 10 µg/mL laminin and 10 µg/mL poly-ornithine. Following cell attachment in DMEM/15% FBS, cells were grown in defined media consisting of DMEM/F12, 1× N2-supplement, 1× penicillin/streptomycin, 0.01 µg/mL biotin, 15 nM T_3_, 0.11 mg/mL sodium pyruvate, 0.5× BSA, 0.5× FBS. Culture medium was changed every 3 days, and cells were grown for 21 days.

### 4.8. Flow Cytometry

Cells were removed from flasks using 0.1% trypsin. HOG, BHK-21, and L2 live cells were washed in PBS containing 10% FBS and 1% sodium azide. Cells were treated with Myaptavin-3064, 3060-BS, or 3202 BS at indicated concentrations (100 nM, 75 nM, 50 nM, 25 nM in oligonucleotide equivalents) for 1 h on ice. DNA aptamers were diluted in PBS and 2% BSA. Cells were washed three times to reduce non-specific and low-affinity binding. To detect bound conjugate, fluorescein-5-isothiocyanate (FITC)-conjugated antibody to streptavidin with dilution of 1:1000 in PBS was added with incubation for 30 min on ice. FITC conjugates were protected from the light at all stages. Cells were washed three times and suspended in 500 μL PBS for immediate analysis by flow cytometry to quantify cell associated FITC fluorescence.

### 4.9. Immunocytochemistry

Live cell staining was performed using 100 nM DNA-aptamers and 10 µg/mL O4 antibody diluted in fresh media, 3% BSA and 5% goat serum for 1 h on ice. After washing three times with media, cells were fixed in 4% paraformaldehyde (PFA) for 25 min at room temperature, washed, and blocked with 1% bovine serum albumin, 0.1% Triton X-100 and 5% goat serum in DPBS free of Ca^2+^ and Mg^2+^. Antibodies against GFAP, neuron-specific beta-III tubulin, MBP and CNPase were diluted in blocking buffer and samples were stained overnight at 4 °C. Cells were washed with PBS and stained for 1 h at room temperature with Dylight-488- or Dylight-594- labelled anti-streptavidin or AlexaFluor^®^ 488 and AlexaFluor^®^ 568 conjugated goat secondary antibodies. Cells were counterstained with DAPI diluted in PBS 1:1000 and imaged with an LSM 780 confocal microscope. TIRF was used to detect DNA-aptamer binding to cell membranes. Fluorescent micrographs were exported as TIF files from Zen software (Zeiss).

## Supplementary Materials

The following is available online at https://www.mdpi.com/1424-8247/13/11/403/s1, Figure S1: Myaptavin-3064 does not bind neurons, astrocytes, or oligodendrocytes obtained from embryonic mixed cortical preparations.
