# Supplementary material for: Remyelination-Promoting DNA Aptamer Conjugate Myaptavin-3064 Binds to Adult Oligodendrocytes In Vitro
Source: Pharmaceuticals (Basel). 2020 Nov 19;13(11):403. doi: 10.3390/ph13110403 (PMC7699424; doi:10.3390/ph13110403)

Remyelination-promoting DNA aptamer conjugate
Myaptavin-3064 binds to adult oligodendrocytes

Supplementary Figure 1: Myaptavin-3064 does not bind neurons, astrocytes, or oligodendrocytes obtained from embryonic mixed cortical preparations. Live staining was performed on unfixed cells using (B, F, J) 100 nM Myaptavin-3064 or 3060-BS (D, H, L) for 1 h and O4 for 15 min (A, C). After washing, cells were fixed and permeabilized and stained for (E, G) neuron-specific beta-III tubulin and (I, K) glial fibrillary acidic protein, (GFAP).

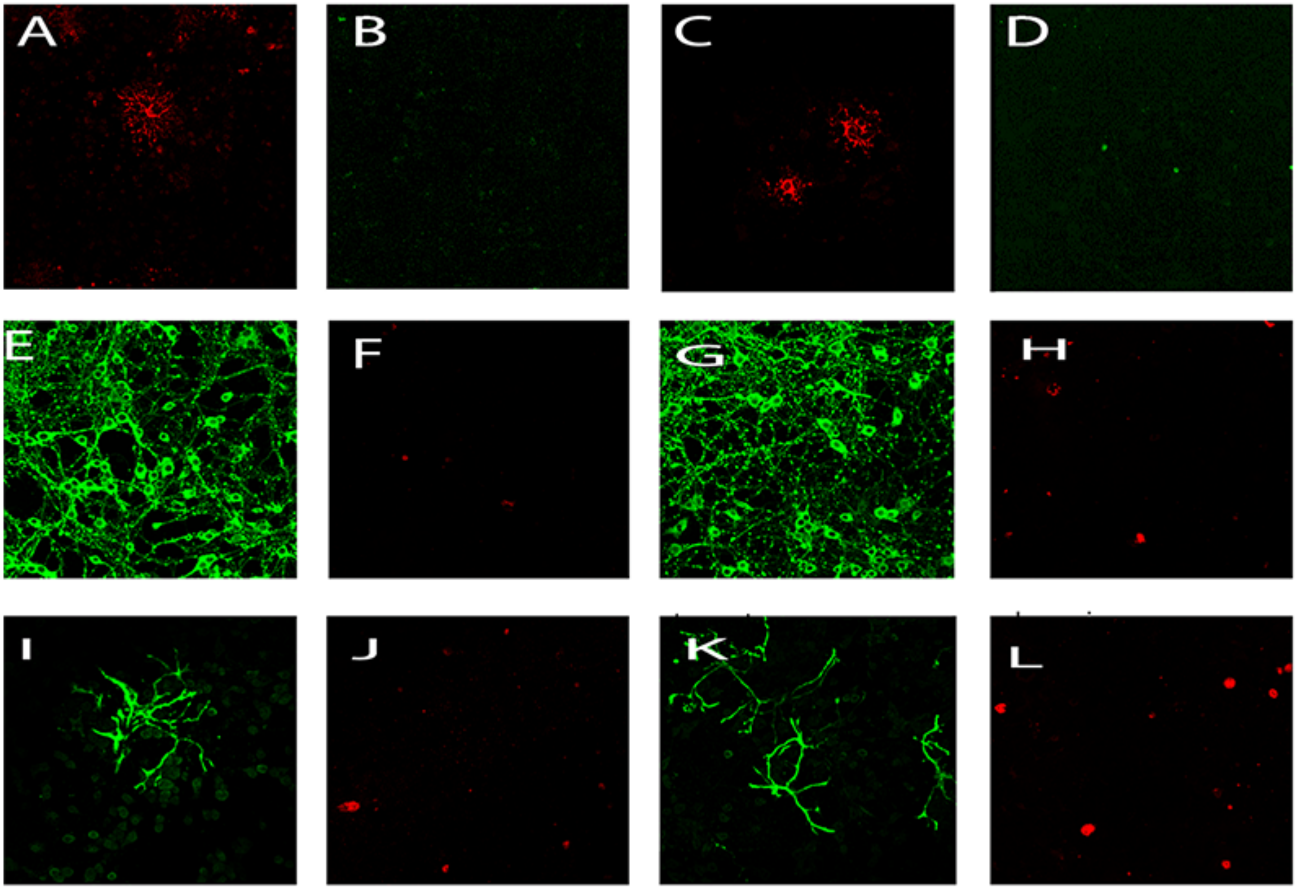

Supplement: Supplementary file 1 [file pharmaceuticals-13-00403-s001.pdf]
